# Availability of Central α4β2* Nicotinic Acetylcholine Receptors in Human Obesity

**DOI:** 10.3390/brainsci12121648

**Published:** 2022-12-01

**Authors:** Eva Schweickert de Palma, Tilman Günnewig, Michael Rullmann, Julia Luthardt, Mohammed K. Hankir, Philipp M. Meyer, Georg-Alexander Becker, Marianne Patt, Sarah Martin, Anja Hilbert, Matthias Blüher, Osama Sabri, Swen Hesse

**Affiliations:** 1 Department of Nuclear Medicine, University of Leipzig, 04013 Leipzig, Germany; 2 Integrated Research and Treatment Center Adiposity Diseases, Leipzig University Medical Centre, 04103 Leipzig, Germany; 3 Max Planck Institute for Human Cognitive and Brain Sciences, 04103 Leipzig, Germany; 4 Department of General, Visceral, Transplantation, Vascular and Pediatric Surgery, University Hospital Wuerzburg, 97080 Wuerzburg, Germany; 5 Department of Psychosomatic Medicine and Psychotherapy, Integrated Research and Treatment Center Adiposity Diseases, Behavioral Medicine Research Unit, University of Leipzig Medical Center, 04103 Leipzig, Germany; 6 Helmholtz Institute for Metabolic, Obesity and Vascular Research (HI-MAG), Helmholtz Zentrum München at the University of Leipzig and University Hospital Leipzig, 04103 Leipzig, Germany; 7 Medical Department III-Endocrinology, Nephrology, Rheumatology, University of Leipzig Medical Center, 04013 Leipzig, Germany; 8 Collaborative Research Centre 1052 Obesity Mechanisms, University of Leipzig, 04103 Leipzig, Germany

**Keywords:** obesity, PET, acetylcholine, nicotinic receptors, (−)-[^18^F]flubatine, nucleus basalis of Meynert, thalamus

## Abstract

Purpose: Obesity is thought to arise, in part, from deficits in the inhibitory control over appetitive behavior. Such motivational processes are regulated by neuromodulators, specifically acetylcholine (ACh), via α4β2* nicotinic ACh receptors (nAChR). These nAChR are highly enriched in the thalamus and contribute to the thalamic gating of cortico-striatal signaling, but also act on the mesoaccumbal reward system. The changes in α4β2* nAChR availability, however, have not been demonstrated in human obesity thus far. The aim of our study was, thus, to investigate whether there is altered brain α4β2* nAChR availability in individuals with obesity compared to normal-weight healthy controls. Methods: We studied 15 non-smoking individuals with obesity (body mass index, BMI: 37.8 ± 3.1 kg/m^2^; age: 39 ± 14 years, 9 females) and 16 normal-weight controls (non-smokers, BMI: 21.9 ± 1.7 kg/m^2^; age: 28 ± 7 years, 13 females) by using PET and the α4β2* nAChR selective (−)-[^18^F]flubatine, which was applied within a bolus-infusion protocol (294 ± 16 MBq). Volume-of-interest (VOI) analysis was performed in order to calculate the regional total distribution volume (V_T_). Results: No overall significant difference in V_T_ between the individuals with obesity and the normal-weight volunteers was found, while the V_T_ in the nucleus basalis of Meynert tended to be lower in the individuals with obesity (10.1 ± 2.1 versus 11.9 ± 2.2; *p* = 0.10), and the V_T_ in the thalamus showed a tendency towards higher values in the individuals with obesity (26.5 ± 2.5 versus 25.9 ± 4.2; *p* = 0.09). Conclusion: While these first data do not show greater brain α4β2* nAChR availability in human obesity overall, the findings of potentially aberrant α4β2* nAChR availability in the key brain regions that regulate feeding behavior merit further exploration.

## 1. Introduction

Obesity is a major public health challenge worldwide. Understanding the processes that contribute to this pandemic is of significant clinical importance as obesity is strongly associated with an increased risk of all-cause morbidity and mortality [1]. The complex physiological process of food intake is necessary for survival and is governed by the homeostatic system and the hedonic systems in the human brain [2]. An imbalance of these two systems can lead to weight gain and obesity [3].

The homeostatic system is located in the hypothalamus, while the hedonic system comprises cortico-limbic-striatal circuits. The main goal of the hypothalamic circuits is to balance the energy intake with the energy expenditure, specifically to balance the food intake with the metabolic needs, whereas the cortico-limbic-striatal circuits cover the affective and pleasurable components of food reward [2,3].

Neurotransmitters, such as dopamine (DA) and serotonin, are crucial for regulating food reward [4]. Brain imaging studies currently suggest lower striatal D2/D3 receptor binding potential in extreme obesity and a higher binding potential in overweight individuals and those with moderate obesity [5], depicting an important but complex role of DA in human obesity.

A neurotransmitter that is known to modulate DA release in the reward system (e.g., the nucleus accumbens (Nac) and the ventral tegmental area (VTA)) is acetylcholine (ACh) [6]. Nicotinic ACh receptors (nAChR) are highly expressed on the dopamine terminals, in the reward circuits, and in the areas of inhibitory control [3]. The thalamus has the highest density of certain subtypes of nAChR [7,8,9]. Nicotine, as an exogenous agonist on AChR, has a strong influence on the appetite, weight control, and food reward. Smokers are known to gain weight when they are quitting [10], while high-restrained eating is associated with elevated rates of smoking compared to the general population [11], and nicotine suppresses the appetite even in healthy non-smoking individuals [12]. Moreover, nicotinic agonists decrease food intake, body mass index (BMI, kg/m^2^), and weight gain by activating the hypothalamic pro-opiomelanocortin (POMC) neuron pathway through the central activation of α3β4* nAChR [13,14].

One of the major subtypes of nAChR that is expressed in the brain, the α4β2* nAChR, has been frequently associated with the mesoaccumbal reward system [3]. Studies have shown that these high-affinity α4β2* nAChR have an effect on the brain circuitries that are involved in reinforcement, mood, attention, and food consumption [15] through modulating DA release in the Nac [6,7]. In addition, the peripheral α4β2* nAChR are known to mediate the effect of nicotine, potentiating satiation and delaying food transit through a vago-vagal reflex [16]. A key role of α4β2* nAChR in both food reward and appetite control, and hence weight gain and the development of obesity, can therefore be assumed.

However, despite this apparently important role in energy balance, in vivo imaging on α4β2* nAChR by using positron emission tomography (PET) and radiotracers that are selective for α4β2* nAChR, such as (−)-[^18^F]flubatine [9] in humans, have not been performed to date. In order to depict the alterations of modulatory α4β2* nAChR availability in the brain regions that are relevant for eating control, an investigation of α4β2* nAChR availability in these brain regions could be a step forward in finding healthy alternatives to smoking for weight control [17].

In this exploratory study, we therefore investigated for the first time in vivo α4β2* nAChR availability in individuals with obesity compared with normal-weight volunteers using PET and the radiotracer (−)-[^18^F]flubatine [9,18]. The regions of interest include the brain areas that are important in eating circuits (NAc, VTA, the orbitofrontal cortex (OFC), the prefrontal cortex (PFC), the amygdala, and the insula), as well as the core regions of the central cholinergic system (nucleus basalis of Meynert (NBM) and the thalamus). We hypothesized that, in these core areas of the basal forebrain and the thalamo-cortical system specifically, the α4β2* nAChR availability is altered in the individuals with obesity compared to the normal-weight healthy controls.

## 2. Materials and Methods

This clinical study was performed according to the 1964 declaration of Helsinki and subsequent revisions and was approved by the local ethics committee (number 225-15-01062015), as well as the national radiation protection agency. The trial is registered at the Deutsches Register für klinische Studien (DRKS) under DRKS00010927.

### 2.1. Study Participants

We consecutively included 15 volunteers with obesity with a BMI of >35 kg/m^2^ and 16 normal-weight volunteers with a BMI of <25 kg/m^2^. All of the study participants were non-smokers, were free for any kind of centrally acting medication, and had no history of neurological or psychiatric illness. They were recruited from public postings and nutritional therapy groups. The exclusion criteria were as follows: neurosurgery in the past, structural tissue lesions, the use of medication or surgery for weight reduction in the last six months, a vegan diet, and contraindications for magnetic resonance imaging (MRI). All of the study participants were asked to complete the Three-Factor Eating Questionnaire (TFEQ, [19]), which measures three dimensions of eating behavior (‘disinhibition’, ‘hunger’, and ‘cognitive restraint’). The normal-weight volunteers with a score that was higher than seven points in the subscale ‘disinhibition’ of the TFEQ were excluded.

### 2.2. PET/MRI Acquisition Protocol and Processing

The (−)-[^18^F]flubatine was synthesized according to a fully automated and GMP-compliant procedure utilizing a commercially available synthesis module (TRACERlab FX FN) [20]. To avoid arterial blood sampling, PET was performed by applying a bolus plus constant infusion (B/I) protocol, according to Hillmer et al. [18], using the PET/MRI Biograph mMR system (Siemens) as follows: after a 90 s intravenous bolus of 202 ± 6 MBq (−)-[^18^F]flubatine, 91 ± 15 MBq (98 mL solution with an hourly rate of 0.6 mL/min) was constantly infused to the end of an in-total scanning time of 165 min, resulting in a total administered activity of 294 ± 16 MBq (−)-[^18^F]flubatine (Table 1). Starting with the bolus injection (minute zero), the dynamic PET data were acquired in list-mode through a standardized high resolution 3D imaging protocol sequentially to 60 post injection (p.i.; 20 frames with 4 × 15 s, 4 × 60 s, 5 × 120 s, 5 × 300 s, and 2 × 600 s) and between 120 and 165 p.i. (13 frames with 12 × 212 s and 1 × 156 s).

The PET data were corrected for attenuation using ultra-short echo time sequences (UTE) [21], scatter, and radioactive decay and were reconstructed into 256 × 256 × 127 matrix with 1.0 × 1.0 × 2.03 mm^3^ voxel size using the built-in 3D ordered subset expectation maximization algorithm with 8 iterations, 21 subsets, and a 3 mm Gaussian filter. The MRI sequences included a high-resolution T1-weigthed 3D magnetization-prepared rapid gradient-echo (MPRAGE) (repetition time: 1900 ms, echo time: 2.53 ms, inversion time: 900 ms) sequence to perform anatomical mapping for the generation of volumes of interest (VOIs) in the PET data sets. UTE sequences were acquired shortly before the radiotracer bolus administration, while the MPRAGE was obtained within the first minutes thereafter.

**Figure 1:**
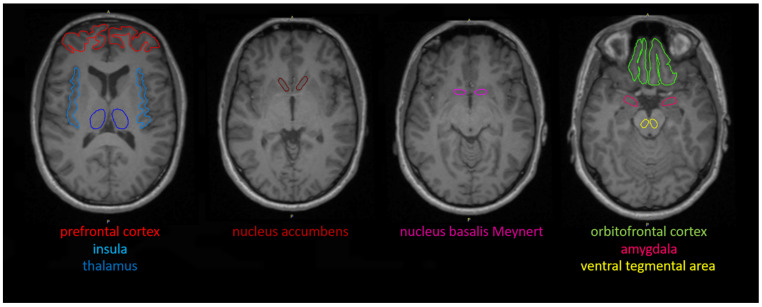
Manually drawn, anatomical-based volumes of interest defined in individual transaxial magnetic resonance imaging data sets (same text color was used for the specific brain region in each slice).

**Figure 2:**
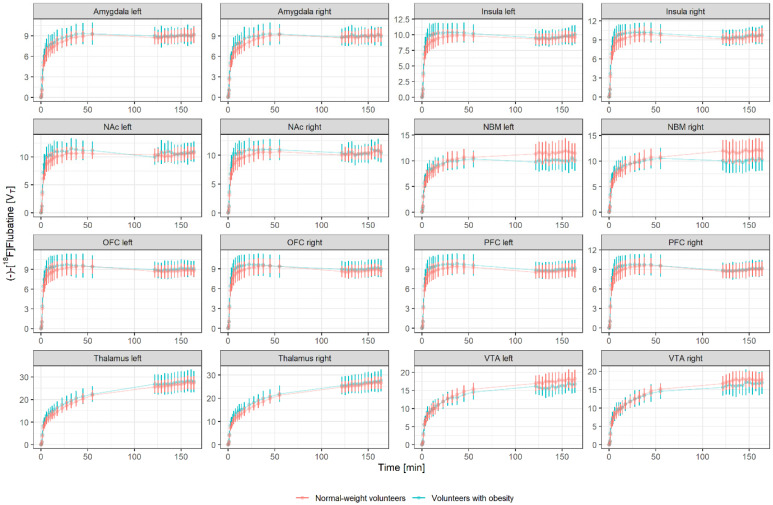
Generated average time activity curves after applying (−)-[^18^F]flubatine in a bolus plus constant infusion protocol of both study cohorts for each volume of interest; first phase from 0 to 60 min p.i. consisting of 20 frames and second phase from 120 to 165 min p.i. consisting of 13 frames.

The PET and MRI data were processed further using PMOD (version 3.5, PMOD Technologies, Zurich, Switzerland). First, eight VOIs were drawn manually on consecutive transversal slices of the individual MRI data sets in the brain areas that are relevant for feeding control and the core regions of the brain cholinergic system (amygdala, insula, NAc, NBM, OFC, PFC, thalamus, and VTA; Figure 1). Then, the PET data were corrected for head motion artifacts and were co-registered with the individual MRI data and the related VOI set to obtain corresponding tissue time activity curves (TACs) from the dynamic PET data (Figure 2).

For the calculation of the total distribution volume (V_T_), venous blood samples at 90 min, 105 min, 120 min, 135 min, 150 min, and 160 min p.i. with four probes of 2 ml at each time point, resulting in a total blood volume of 48 mL (6 × 4 × 2 mL), were taken. The 120–165 min p.i. tracer concentration in tissue (C_tissue_) at equilibrium were divided by the total radioactivity concentration in the venous plasma (C_plasma_) to generate the total distribution volume (V_T_) for each VOI. For an optimal bias-variance tradeoff, metabolite correction was not considered in the present analysis. This is because our own previous pilot data using the arterial input function without metabolite correction yielded only slightly (about 10%) lower distribution volumes for all regions, since the uncorrected input function at 90 min is about 10% higher. Nevertheless, the relative standard deviation of V_T_ was lower with the input functions without metabolite correction [9].

### 2.3. Statistical Analyses

The statistical analyses were performed with Jamovi 1.6.23. A Student’s *t*-test and exact Fisher test (significance at *p* < 0.05; Table 1) were performed to test for group differences. As ‘age’ differed between the normal-weight volunteers and the individuals with obesity, we used ANCOVA with age as a covariate to compare the V_T_ of both study groups. The correlation of V_T_ with subscales of TFEQ separately for participants with obesity and normal-weight participants were analyzed through partial correlation controlling for age.

## 3. Results

Table 1 shows the demographic data of the study participants. The age was significantly higher in the individuals with obesity compared with the normal-weight volunteers, while the TFEQ scores of ‘disinhibition’ and ‘hunger’ were significantly lower in the normal-weight controls compared to the individuals with obesity.

**Figure 3:**
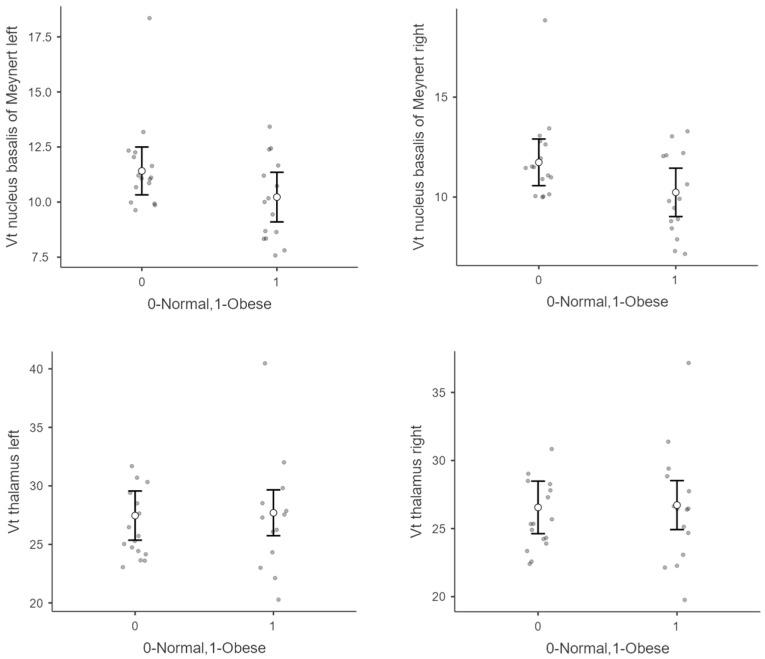
Total distribution volume (V_T_) of (−)-[^18^F]flubatine in individuals with obesity and normal-weight controls in the right and left nucleus basalis of Meynert and the right and left thalamus shown as Jitter plots. The mean value is depicted with the white point and error bars representing the standard deviation. Individual data points are represented in gray.

**Figure 4:**
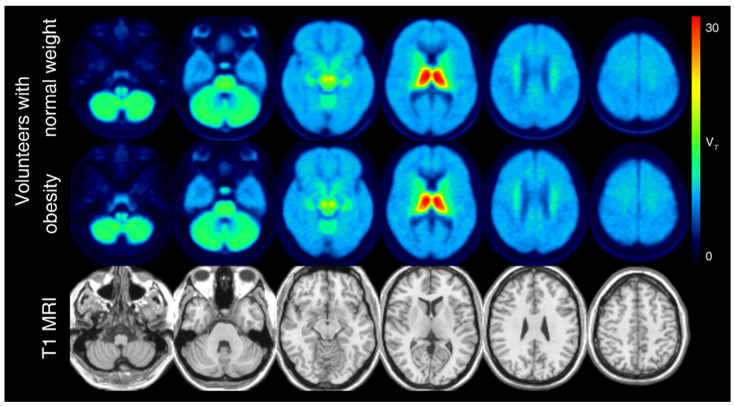
The (−)-[^18^F]flubatine average total distribution volume of all normal-weight volunteers (first row) and volunteers with obesity (second row) using positron emission tomography co-registered to T1 (third row) magnetic resonance imaging.

Table 2 presents the V_T_ in the individuals with obesity and the normal-weight volunteers, controlled for age. Overall, no significant differences in V_T_ between the individuals with obesity and the normal-weight controls were found. The V_T_ in the NBM tended to be lower in the volunteers with obesity compared with the normal-weight volunteers (Table 2, Figure 3), while the V_T_ in the thalamus showed a tendency towards higher values in the volunteers with obesity versus the normal-weight volunteers (Table 2, Figure 3). The mean V_T_ of the normal-weight volunteers and the volunteers with obesity is shown in Figure 4.

**Figure 5:**
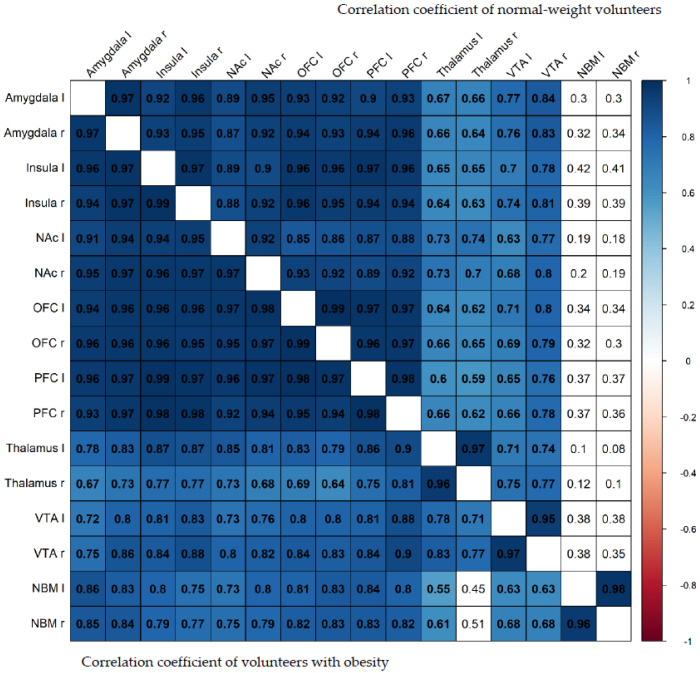
Correlation heatmap showing the strength of correlations between all volumes of interest, while blue indicates a positive correlation and red a negative correlation (*p* < 0.05). Fields with a white background represent non-significant correlations (*p* > 0.05). A partial correlation of V_T_ with subscales of the TFEQ performed separately for individuals with obesity and normal-weight controls showed that none of the TFEQ subscales correlated significantly with V_T_ (Table 3).

A correlation heatmap of all of the VOIs is shown in Figure 5. The V_T_ of the NBM appears to be less associated with the V_T_ of all of the other regions, particularly in the normal-weight controls, while the interregional correlations seem to be stronger in the volunteers with obesity versus the normal-weight volunteers.

## 4. Discussion

This study investigated for the first time in vivo α4β2* nAChR availability in the brain regions that are important in feeding circuits in volunteers with obesity compared to normal-weight volunteers. Overall, no significant differences in α4β2* nAChR availability were found between the study groups. However, a tendency for lower V_T_ in the NBM in the volunteers with obesity, as well as a tendency for higher V_T_ in the thalamus, was found.

Besides the hypothalamus and the brainstem, the thalamus serves as an important independent input region for cholinergic signaling and functions [22]. Through cholinergic neuromodulation, the thalamus responds to external cues [23] and has the highest density of α4β2* nAChRs [7,8,9]. It is one of the most important brain regions modulating attentional control [24,25]. An example of this is given in the computational modeling of Schmitt et al. [25] as follows: enhanced mediodorsal thalamic (MD) excitability increased the PFC rule information content by improving the tuning of individual cortical neurons and also by recruiting the previously untuned cortical neurons. A thalamic circuit such as the MD therefore amplifies the local cortical connectivity in order to sustain the attentional control. A higher receptor availability in the volunteers with obesity than in the normal-weight volunteers in this region could hint to a higher receptiveness towards incoming cues, in this case food stimuli. Regarding the receptor dynamics, preliminary data applying stimulation indicated that the α4β2* nAChR availability underlies the changes in the response to high-salient food cues [26]. Further and more extensive investigations, including studies that explore α4β2* nAChR availability with stimulation designs and functional MRI, are needed in order to verify these tendencies.

As one of the extrathalamic inputs, and the most extensive, cholinergic afferents center in the NBM from where they are distributed throughout the cerebral cortex [7,27]. According to these widespread projections, the cholinergic modulation of the NBM has been implicated to play an important role in distinct neurobehavioral functions, such as attention, arousal, memory, and food intake [13,15,28]. The tendency of lower V_T_ in this region in the volunteers with obesity may hint to an altered cholinergic signaling, leading to increased food intake [29].

Paolone et al. [30] indicated impaired cholinergic stimulation of cortical circuitry through α4β2* nAChRs that were prone to attribute high-incentive salience to reward cues and poor attentional control as a vulnerability factor for obesity. Interestingly, α4β2* nAChR availability of the NBM was less correlated with those of all of the other brain regions, particularly in the normal-weight volunteers, while, in general, α4β2* nAChR availability appears to be more related to each other, probably indicating higher cholinergic tone in the volunteers with obesity.

The pathophysiological meaning of the putative differences in the α4β2* nAChRs is less clear. This could represent receptor desensitization, i.e., a loss of response after prolonged or repeated application of a stimulus [31], similar to tobacco use. In addition, constant stimulation of nAChRs in nicotine addiction can lead to receptor desensitization [32,33,34]. A tendency for lower nAChR availability in the volunteers with obesity could suggest a nAChR desensitization as an expression of a dysfunctional NBM.

In contrast, Buisson and Betrand [34] saw “hyperfunctional” α4β2* nAChRs after nicotine removal, even with an overall higher apparent affinity for ACh and currents of higher amplitudes with less desensitization. It seems that activation, as well as desensitization, contribute to the rewarding properties of nicotine inside of the cholinergic system [32].

With regard to the clinical implications, our investigation of α4β2*nAChRs goes beyond the current targets, such as dopamine and serotonin, and gives a perspective as a future molecular target for treating human obesity.

## 5. Limitations

These first data did not detect significant changes in α4β2* nAChR availability in obesity. This could be due to the relatively low number of participants included in the study. By applying the V_T_ results in the right NBM (difference of means 1.8, standard deviation 2.2) that were obtained in this study, we estimated a necessary sample size of 24 participants in each group for an independent *t*-test (alpha = 0.05, power = 0.8; power and sample size calculations; PS version 3.1.2; [35]) for future cross-sectional studies. Furthermore, we cannot estimate the time scales of the changes in α4β2* nAChR availability given the nature of these fast acting, allosteric receptors; rapid, dynamic interactions may also interfere at the individual signal level.

## 6. Conclusions

This is the first study on α4β2* nAChR that has used PET imaging in vivo and the high-affinity selective radiotracer (−)-[^18^F]flubatine to study human obesity. Overall, these first data showed no statistically significant V_T_ differences between the individuals with obesity and the normal-weight controls. However, these data also revealed trends toward lower α4β2* nAChR availability in the nucleus basalis of Meynert and higher α4β2* nAChR availability in the thalamus. Whether these tendencies become relevant in modulating eating behavior needs to be further explored, including studies that explore α4β2* nAChR availability with stimulation designs in order to define α4β2* nAChR as a promising target for future intervention.

## Figures and Tables

**Table 1:** Demographics and Three-Factor Eating Questionnaire scores of individuals with obesity and normal-weight controls.

Variables	Individuals with Obesity (*n* = 15)	Normal-Weight Controls (*n* = 16)	*p* Value
Age (years)	39.5 ± 14.0 (20 to 62)	27.5 ± 7.3 (19–45)	0.005 ^a^
Sex (male/female)	6/9	3/13	0.25 ^b^
BMI (kg/m^2^)	37.8 ± 3.1(32.5 to 42.8)	21.9 ± 1.7 (19.4 to 24.9)	<0.001 ^a^
Activity (MBq)	291 ± 8.5 (270 to 300)	295 ± 8.0 (273 to 307)	0.15 ^a^
TFEQ score			
Cognitive control	8.7 ± 5.5 (1 to 19)	7.2 ± 3.9 (2 to 15)	0.37 ^a^
Disinhibition	8.9 ± 3.6 (3 to 14)	3.4 ± 2.1 (0 to 7)	<0.001 ^a^
Hunger	5.6 ± 2.6 (2 to 9)	3.2 ± 2.8 (0 to 10)	0.02 ^a^

Mean ± SD (range); ^a^ Student’s *t*-test; ^b^ exact Fisher test; SD: standard deviation; BMI: body mass index; TFEQ: Three-Factor Eating Questionnaire.

**Table 2:** Comparison of total distribution volumes (V_T_) in regions of interest between individuals with obesity and normal-weight controls.

		Individuals with Obesity (*n* = 15)		Normal-Weight Controls (*n* = 16)		
Region		Mean	SD	Mean	SD	*p* ^a^
Amygdala	Left	9.00	1.2	8.91	0.9	0.55
	Right	8.99	1.3	8.93	0.9	0.66
Insula	Left	9.63	1.3	9.49	0.9	0.40
	Right	9.55	1.3	9.40	0.9	0.44
Nucleus accumbens	Left	10.60	1.7	10.40	1.0	0.62
	Right	10.40	1.5	10.40	1.0	0.70
Nucleus basalis of Meynert	Left	10.10	1.9	11.60	2.1	0.16
	Right	10.10	2.1	11.90	2.2	0.10
Orbitofrontal cortex	Left	9.03	1.3	8.77	0.9	0.46
	Right	8.97	1.3	8.68	0.9	0.42
Prefrontal cortex	Left	8.88	1.3	8.67	1.1	0.35
	Right	8.96	1.2	8.87	1.1	0.43
Thalamus	Left	27.40	4.7	26.50	2.8	0.15
	Right	26.50	4.2	25.90	2.5	0.09
Ventral tegmental area	Left	16.10	2.2	17.60	2.2	0.38
	Right	16.40	2.6	17.50	2.1	0.61

^a^ ANCOVA with age as covariate.

**Table 3:** Partial correlation of total distribution volume (V_T_) with subscales of the Three-Factor Eating Questionnaire (cognitive control, disinhibition, and hunger) in individuals with obesity (*n* = 15) and in normal-weight controls (*n* = 16).

			Individuals with Obesity (*n* = 15)			Normal-Weight Controls (*n* = 16)		
Region			Control	Disinhibition	Hunger	Control	Disinhibition	Hunger
Amygdala	Left	r	−0.125	−0.114	0.251	0.127	0.327	0.137
		p	0.460	0.698	0.387	0.653	0.235	0.628
	Right	r	−0.261	0.007	0.333	0.186	0.292	0.191
		p	0.367	0.982	0.245	0.506	0.292	0.495
Insula	Left	r	−0.241	0.087	0.432	0.124	0.194	0.160
		p	0.406	0.767	0.123	0.660	0.488	0.569
	Right	r	−0.254	−0.005	0.403	0.150	0.289	0.200
		p	0.381	0.987	0.153	0.594	0.297	0.475
Nucleus accumbens	Left	r	−0.375	−0.015	0.320	−0.011	0.278	0.153
		p	0.186	0.959	0.265	0.970	0.315	0.587
	Right	r	−0.319	0.077	0.382	0.183	0.483	0.362
		p	0.266	0.793	0.178	0.514	0.068	0.185
Nucleus basalis of Meynert	Left	r	−0.274	0.047	0.219	−0.150	0.276	0.117
		p	0.344	0.873	0.452	0.595	0.320	0.679
	Right	r	−0.257	0.080	0.275	−0.047	0.119	0.067
		p	0.374	0.785	0.342	0.868	0.673	0.811
Orbitofrontal cortex	Left	r	−0.318	−0.045	0.380	0.320	0.321	0.245
		p	0.268	0.879	0.180	0.244	0.243	0.379
	Right	r	−0.307	−0.065	0.408	0.310	0.310	0.272
		p	0.286	0.825	0.148	0.260	0.260	0.326
Prefrontal cortex	Left	r	−0.347	0.142	0.467	0.225	0.263	0.205
		p	0.225	0.628	0.092	0.350	0.344	0.464
	Right	r	−0.279	0.068	0.449	0.223	0.374	0.170
		p	0.334	0.819	0.108	0.424	0.170	0.545
Thalamus	Left	r	−0.491	−0.193	0.144	0.306	0.043	0.079
		p	0.334	0.509	0.623	0.267	0.89	0.781
	Right	r	−0.356	0.007	0.153	0.236	0.020	0.048
		p	0.211	0.980	0.602	0.396	0.944	0.865
Ventral tegmental area	Left	r	−0.291	0.061	0.523	0.210	0.038	0.183
		p	0.313	0.836	0.055	0.452	0.892	0.515
	Right	r	−0.300	0.015	0.449	0.257	0.118	0.285
		p	0.298	0.960	0.107	0.356	0.675	0.302

Spearman’s rho Note: controlling for age.

## Data Availability

The data presented in this study are available upon request from the corresponding author. The data are not publicly available due to the fact that they contain information that could compromise the privacy of the participants.
